# Development of Implant/Interconnected Porous Hydroxyapatite Complex as New Concept Graft Material

**DOI:** 10.1371/journal.pone.0049051

**Published:** 2012-11-09

**Authors:** Kazuya Doi, Hiroshi Oue, Koji Morita, Shiho Kajihara, Takayasu Kubo, Katsunori Koretake, Vittoria Perrotti, Giovanna Iezzi, Adriano Piattelli, Yasumasa Akagawa

**Affiliations:** 1 Department of Advanced Prosthodontics, Hiroshima University Graduate School of Biomedical Sciences, Hiroshima, Japan; 2 Department of Medical, Oral and Biotechnological Sciences, Chieti-Pescara University, Chieti, Italy; University of Notre Dame, United States of America

## Abstract

**Background:**

Dental implant has been successfully used to replace missing teeth. However, in some clinical situations, implant placement may be difficult because of a large bone defect. We designed novel complex biomaterial to simultaneously restore bone and place implant. This complex was incorporated implant into interconnected porous calcium hydroxyapatite (IP-CHA). We then tested this Implant/IP-CHA complex and evaluated its effect on subsequent bone regeneration and implant stability *in vivo*.

**Methodology/Principal Findings:**

A cylinder-type IP-CHA was used in this study. After forming inside of the cylinder, an implant was placed inside to fabricate the Implant/IP-CHA complex. This complex was then placed into the prepared bone socket in the femur of four beagle-Labrador hybrid dogs. As a control, implants were placed directly into the femur without any bone substrate. Bone sockets were allowed to heal for 2, 3 and 6 months and implant stability quotients (ISQ) were measured. Finally, tissue blocks containing the Implant/IP-CHA complexes were harvested. Specimens were processed for histology and stained with toluidine blue and bone implant contact (BIC) was measured. The ISQs of complex groups was 77.8±2.9 in the 6-month, 72.0±5.7 in the 3-month and 47.4±11.0 in the 2-month. There was no significant difference between the 3- or 6-month complex groups and implant control groups. In the 2-month group, connective tissue, including capillary angiogenesis, was predominant around the implants, although newly formed bone could also be observed. While, in the 3 and 6-month groups, newly formed bone could be seen in contact to most of the implant surface. The BICs of complex groups was 2.18±3.77 in the 2-month, 44.03±29.58 in the 3-month, and 51.23±8.25 in the 6-month. Significant difference was detected between the 2 and 6-month.

**Conclusions/Significance:**

Within the results of this study, the IP-CHA/implant complex might be able to achieve both bone reconstruction and implant stability.

## Background

The use of dental implants has become widespread as prosthetic therapy for patients with missing teeth. The success of an implant relies on the presence of adequate bone quantity and quality at the placement site because the implant needs to undergo “osseointegration” [1]. However, in some clinical situations, the condition of bone is not always optimal to allow the implant to be stably integrated into the placement site. In such cases, bone augmentation, including guided bone regeneration, has received considerable attention [2], [3], [4], [5].

In a large bone defect is caused by injury or tumor, implant treatment becomes more difficult. Moreover, if the functional implant site becomes infected, leading to peri-implantitis, the circumference of the affected bone in contact with the implant is resorbed, resulting in enlargement of the surrounding area owing to an inflammatory reaction. In such cases, the implant and circumference of the affected bone require surgical removal, resulting in a large bone defect, which allows implant treatment only after bone reconstruction using some bone grafts, however, bone augmentation could be a tricky procedure, healing period may be very long, multiple surgeries are usually required, with a consequent increased risk of morbidity for patients due to the multiple step approach. In particular, when implants are not placed simultaneously to bone augmentation procedures a further decrease in the bone gain should be expected. Therefore, growing need to develop a novel approach to simultaneously restore bone tissue and place implant exists. In our knowledge, there is no reports evaluated such approach. Recently, interconnected porous calcium hydroxyapatite (IP-CHA) ceramics have been introduced for use as scaffold for bone regeneration [6], and are now widely used in the clinical field [7], [8], [9], [10]. IP-CHA has a systematic arrangement of spherical uniform pores with interconnections and has been reported to be successfully used as bone-grafting material in the field of tissue engineering. Also, there are some reports that biomaterials of porous structure had applied for implant graft material [11], [12]. Previous studies conducted in our laboratory demonstrated that both of cell-hybrid artificial bone where bone marrow stromal calls were injected to IP-CHA and polyphosphate adsorbed IP-CHA could enhance bone formation [13], [14], [15], [16].

**Figure 1:**
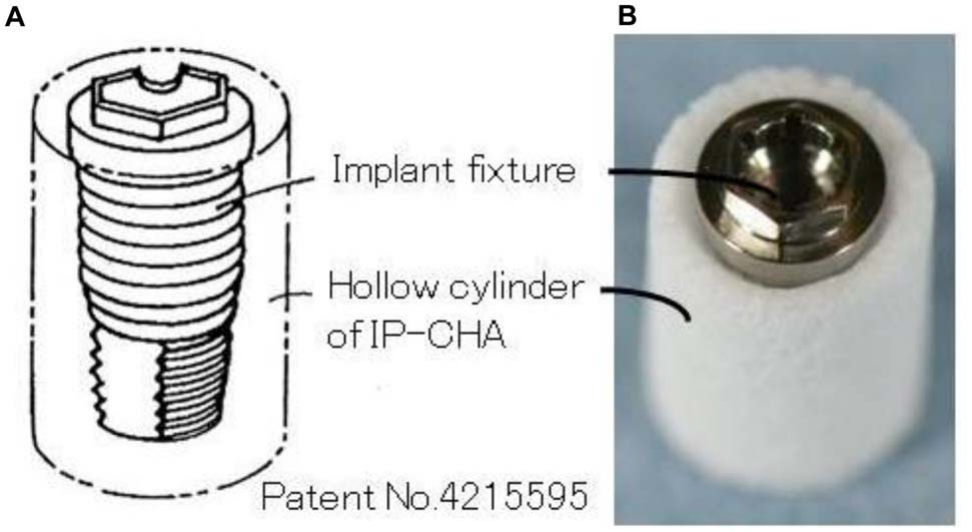
Implant/interconnected porous calcium hydroxyapatite (IP-CHA) Complex. (A) Outline (B) fabricated complex, showing the assembled graft consisting of the IP-CHA and implant fixture.

Basing on the specific characteristic of the IP-CHA, we designed a novel original complex to be used as graft material (Fig. 1A). Specifically, the complex consists of dental implant fixture surrounded by a hollow block of IP-CHA, which is thought to enhance osteoconduction and improve implant stability. The hypothesis is that the complex replaces lost bone tissue, supports new bone formation and allows a simultaneous implant placement with a shortening of the time and surgical steps. To the best of our knowledge, the application of such a complex has hitherto not been reported. This complex is expected to be useful in the fields of dentistry and orthopedics as a novel, simplified approach to achieve bone rehabilitation. Therefore, the aim of the present study was to test the implant/IP-CHA complex developed and to assess its effects on subsequent bone regeneration and implant stability in an animal model.

## Materials and Methods

### Materials

**Figure 2:**
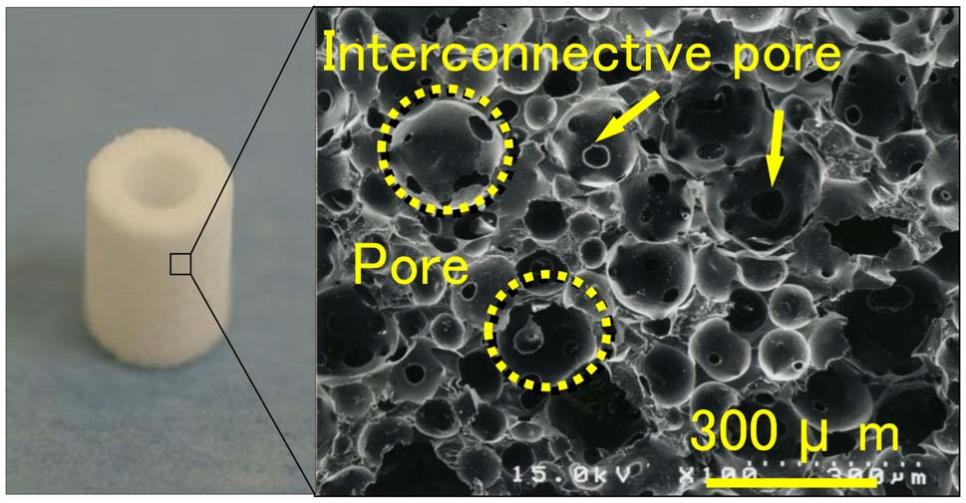
IP-CHA fabricated of a hollow cylinder. (A) Photograph of the prepared IP-CHA (B) A SEM image of the surface. IP-CHA has a systematic arrangement of uniform pores, all of which are connected by a network of smaller interconnected pores.

The hydroxyapatite (HA) used in this study was custom-fabricated into a hollow cylinder of IP-CHA (outer diameter: 6 mm, inner diameter: 3 mm, height: 8.5 mm)(NEOBONE®; Covalent Materials, Tokyo, Japan) with 75% porosity, and a mean porous diameter of 150 µm, and all pores were connected by interconnected pores of 40 µm diameter (Fig. 2). The IP-CHA structure was manufactured using the “form-gel” technique [6]. The implants used were commercially available pure titanium implants (diameter: 3.75 mm, length: 8.5 mm; Brånemark System® Mk III, Nobel Biocare, Sweden).

### Fabrication of the Implant/IP-CHA Complex

**Figure 3:**
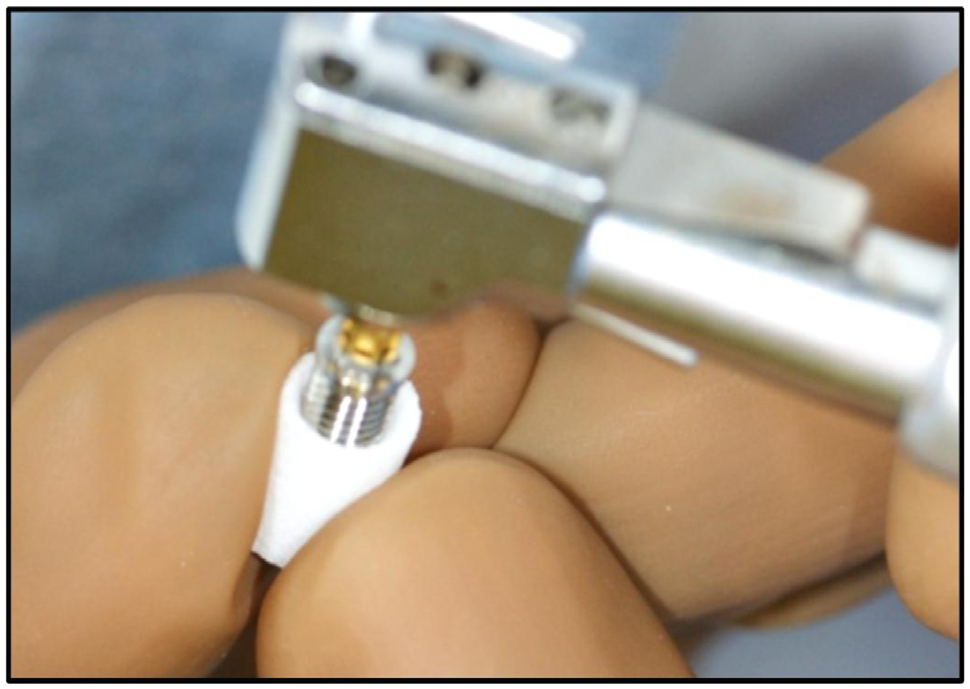
In corporation of a titanium implant into the prepared IP-CHA. After preparation of the IP-CHA, commercially available implants were incorporated into the IP-CHA to fabricate the graft complex. This process was performed under sterile condition.

Fabrication of the complex was performed as follows: first, a screw thread was prepared at the inner surface of a hollow cylinder of IP-CHA using a special electric engine (Nobel Biocare Japan Inc, Tokyo, Japan) with serial cutting drills and a screw tap (Nobel Biocare Japan Inc), and then, countersinking was performed at upper section. After preparation of the IP-CHA cylinder, the titanium implant was incorporated into it to fabricate the complex (Fig. 3). This procedure was performed in accordance to with the Brånemark® system manual.

### Scanning Electron Microscopy Image of the Prepared Cylindrical IP-CHA

After the screw tap and countersink preparation, before incorporation of the implant, one of the hollow IP-CHA cylinders was bisected, and the inner portion was observed by scanning electron microscopy (SEM, JMS-7300, Nihon Denshi Oyo, Tokyo, Japan).

### Observation of the Implant/IP-CHA Complex

One of the Implant/IP-CHA complex was dehydrated using increasing concentrations of ethanol, cleared with styrene monomer, and then embedded in light-polymerized polyester resin (Technovit 7200VLC; Kulzer, Wehrheim, Germany). To achieve complete polymerization of the resin block, photopolymerization equipment was used (BS5000; EXAKT APARATEBAU, Hamburg, Germany). The resin block was cut using a diamond saw system at the center of the Implant/IP-CHA complex, and the cross-section was observed.

### Animal Experiments

The animal research protocol was in accordance with the current version of the Japan Law on the Protection of Animals. This study was approved by the Research Facilities Committee for Laboratory Animal Science at the Hiroshima University School of Medicine, Hiroshima, Japan. All surgery was performed under general anesthesia, and all efforts were made to minimize suffering during experimental period.

**Figure 4:**
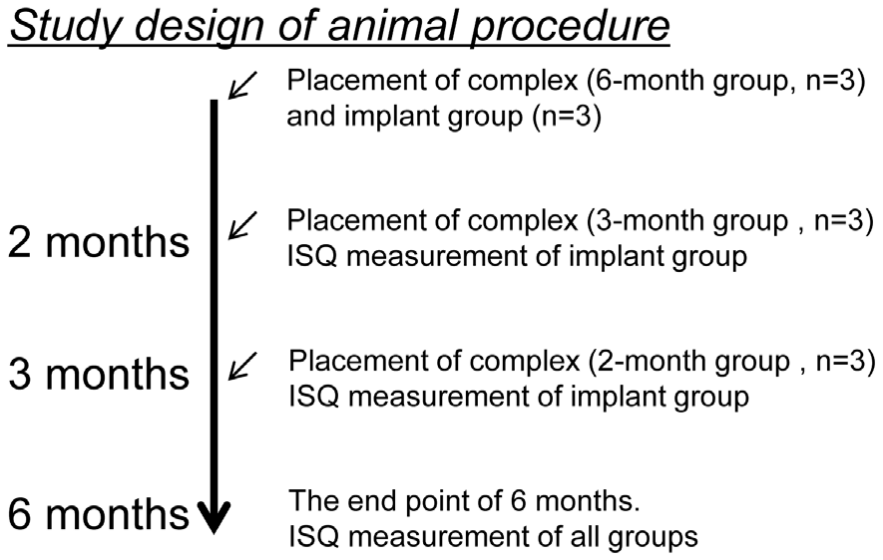
Design of the animal experiment. Three animals were used in this study. Both control implants and test complexes were placed and evaluated over 6 months period. The ISQ of the control implant group was measured continually and the ISQs of the complex groups are measured at the end point.

**Figure 5:**
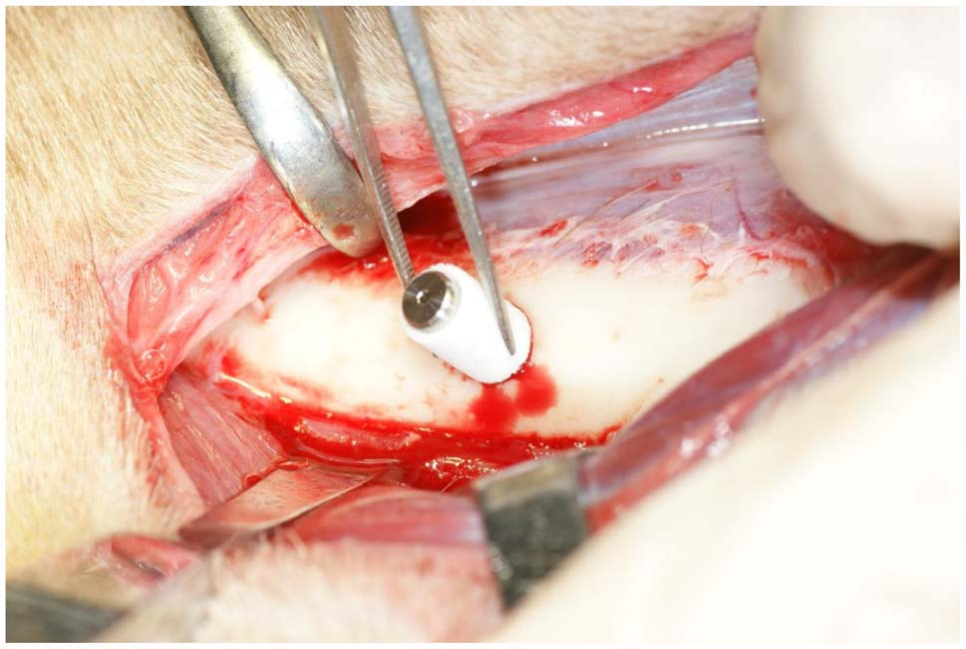
Placement of the Implant/IP-CHA complex. Placement of the graft complex in to a bone sockets in the femur.

The study design of the animal procedure is shown in Fig. 4. Three male Beagle-Labrador hybrid dogs weighing 20–25 kg and aged 20–24 months were fed in their cages for 1 month to allow them to acclimatize to the environment. The Implant/IP-CHA complexes were placed into prepared bone sockets (diameter: 6 mm, height: 10 mm) in the center of the left femur of each of the 3 dogs (Fig. 5). Additionally, titanium implants without IP-CHA were placed beside the Implant/IP-CHA complex as a control. All surgical procedures were performed under general anesthesia with sodium pentobarbital (40 mg/kg) and local infiltration anesthesia with 2% lidocaine and 1∶80,000 noradrenaline. The animals were sacrificed under deep anesthesia and perfused with 10% neutral formalin through the aorta, and the femurs were dissected. Samples were retrieved at different time points (2, 3, and 6 months) and processed to obtain histological sections. Finally, the Implant/IP-CHA complex at 2, 3, and 6 months and the control Implants were set in position (n = 3). The placement design is shown in Table 1.

**Table 1:** Placement part of implants and Implant/IP-CHA complexes.

	Right side		Left side	
**Animal A**	Implant	Complex:6M	Complex:3M	Complex:2M
**Animal B**	Implant	Complex:3M	Complex:2M	Complex:6M
**Animal C**	Implant	Complex:2M	Complex:3M	Complex:6M

### Histological Evaluation

The specimens were immediately fixed in 10% buffered formalin and processed to obtain thin ground sections. Tissue blocks containing implants and Implant/IP-CHA complex were dehydrated using ascending concentrations of ethanol, cleared with styrene monomer, and then embedded in light-polymerized polyester resin (Technovit 7200VLC, Kulzer). To achieve complete polymerization of the resin block, photo polymerization equipment was used (BS5000; EXAKT APARATEBAU). After polymerization, the specimens were sectioned with a high-precision diamond disc to produce a 200-µm-thick cross section. The undecalcified specimens were ground to approximately 70-µm-thick sections (MG5000; EXAKT APPARATEBAU), which were stained with toluidine blue. A light microscope was used for histological examination of the Implant/IP-CHA complex specimens.

### Measurement of Implant Stability Quotient

**Figure 6:**
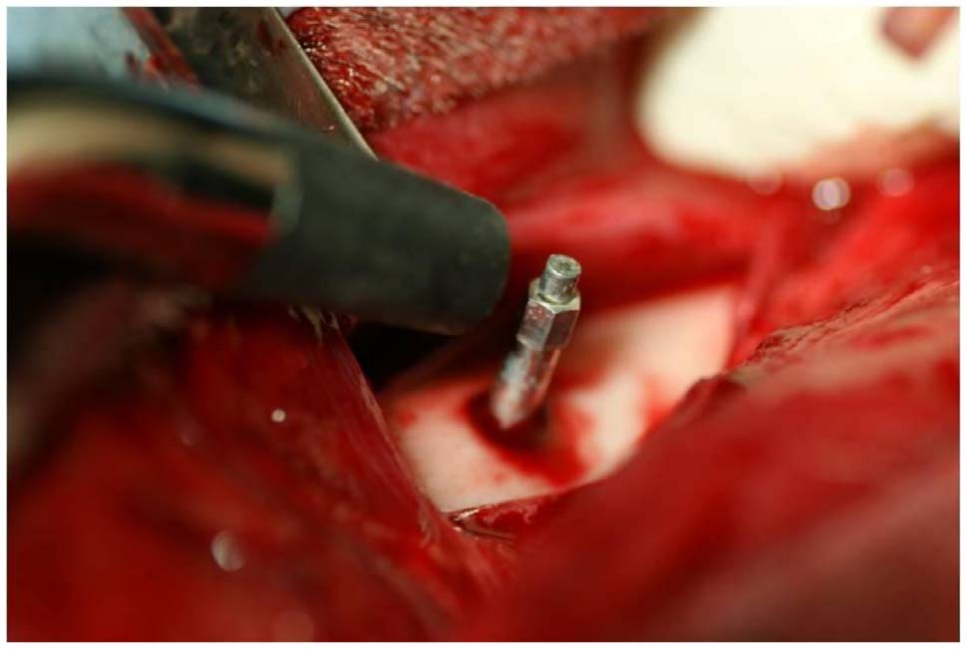
Measurement of ISQ. Stability was measured in the implant control group and the complex groups using an Ostell ISQ™ as RFA.

The implant stability quotient (ISQ) of implants was measured repeatedly at 2, 3 and 6 months. ISQs of Implant/IP-CHA complexes were measured at 6 months ISQ was assessed using Ostell ISQ™ (Ostell Mentaor, Göteborg, Sweden) as resonance frequency analyzer (Fig. 6). After 2, 3, and 6 months placement, the ISQs of all samples was measured.

### Measurement of Bone Implant Contact (BIC) Ratio

The ratio of BIC was measured as the ratio of contact length of newly formed bone in total length from the bottom of cortical bone detected to the top of implant shoulder part [17]. This measurement was performed in the cortical bone area. BIC were measured using NIH Image J (National Institutes of Health, Bethesda, MD).

### Statistical Analysis

The data obtained were expressed as means +/− standard deviations.

The values obtained were statistically analyzed using one-way analysis of variance and Tukey’s HSD test for multiple comparisons, with significance level set at 5%.

## Results

### SEM Image of Formed IP-CHA

**Figure 7:**
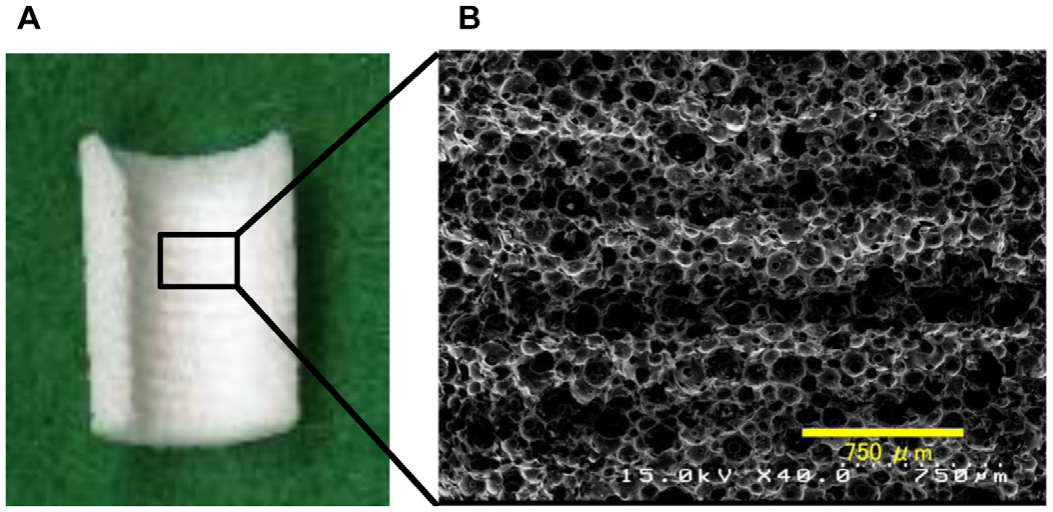
Inner surface of the formed IP-CHA. (A) Photograph of the inner. (B) SEM image of the formed IP-CHA. The shape of the formed screw-thread is evident. After preparation, the characteristic structure was not destroyed, and the interconnected pores remained intact.

Fig. 7 shows an SEM image of the inner surface of the IP-CHA cylinder. The shape of the formed screw-thread is evident. After preparation of the screw-thread, the characteristic structure of IP-CHA could still be seen, and the interconnected pores remained intact.

### Observation of the Implant/IP-CHA Complex

**Figure 8:**
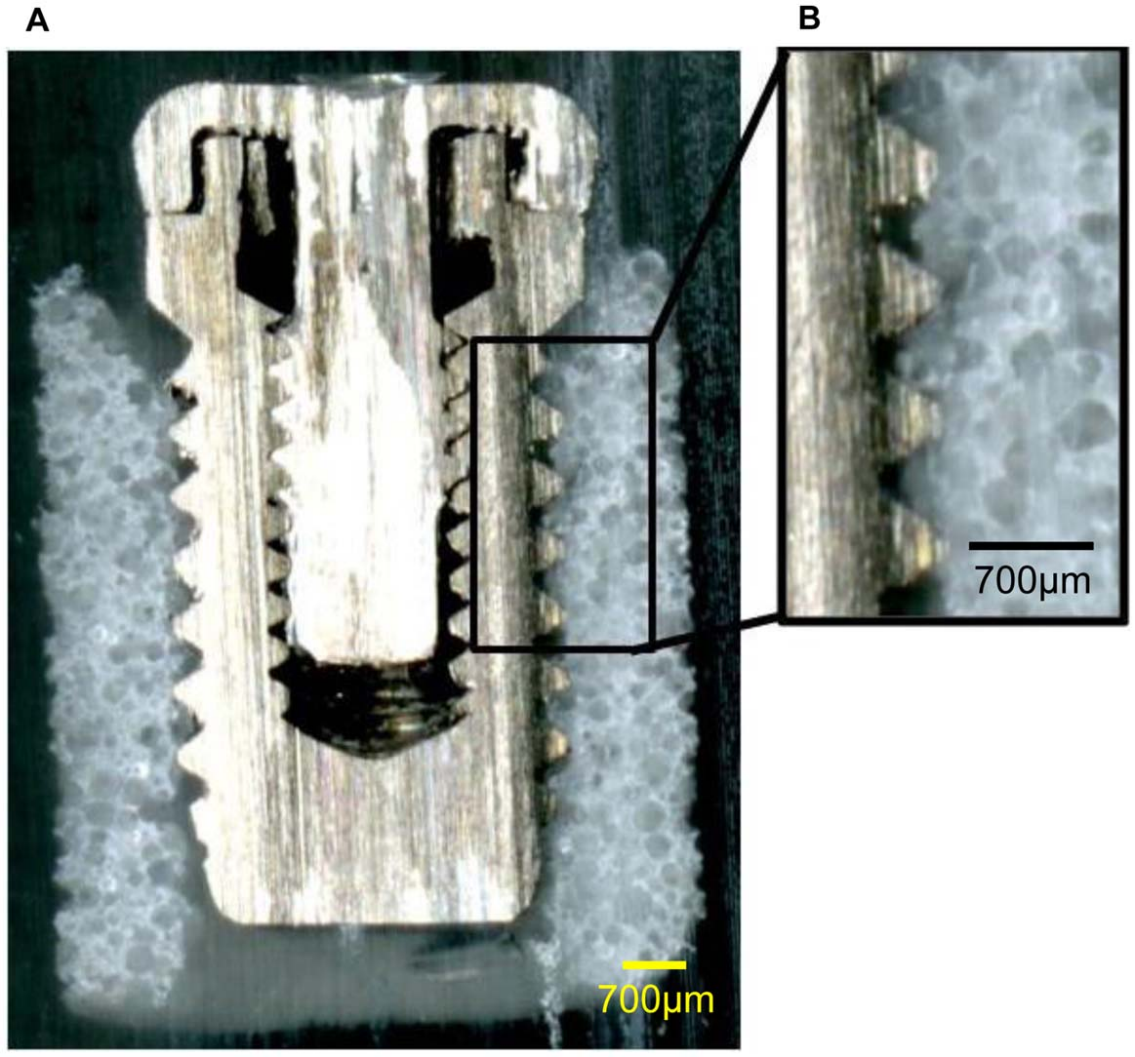
Cross-sectional shape of the Implant/IP-CHA complex. Observation of the cross section of the complex, showing that the implant screw threads and the formed IP-CHA are almost fully3 integrated and there is very little space between the implant threads and the formed IP-CHA.

Fig. 8 shows the central part of the Implant/IP-CHA complex. The implant screw threads and formed IP-CHA were almost fully integrated, and there was very little space between the screw threads and IP-CHA.

### Histological Evaluation

**Figure 9:**
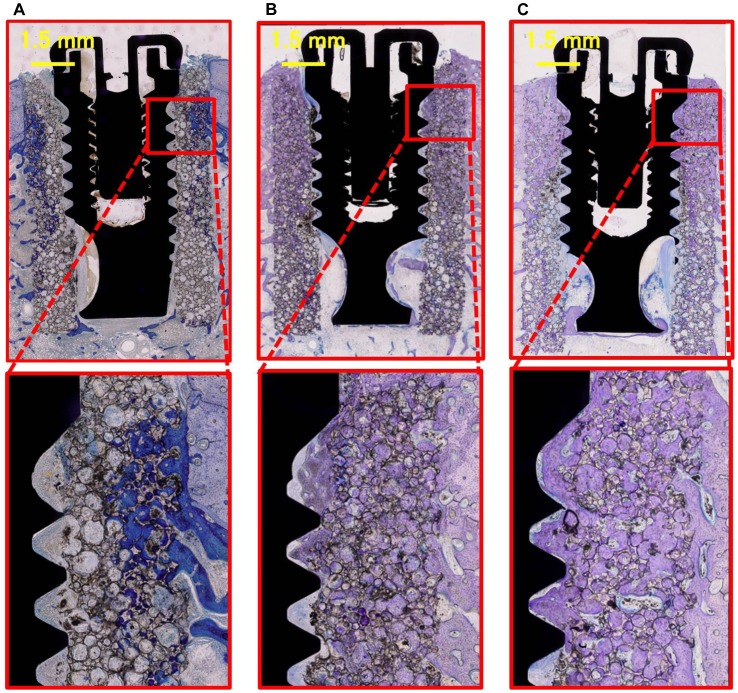
Histology of the Implant/IP-CHA complex groups. (A) Specimen of the 2-month complex, showing newly formed bone, connective tissue and bone marrow detected in the IP-CHA part of the cortical area. At the implant interface, no newly formed bone is observed and the apace is occupied mainly by connective tissue and bone marrow. Therefore, osseointegration is not achieved. (B) Specimen of the 3-month complex, newly formed bone is detected not only in the IP-CHA part but also at the implant interface. Newly formed bone is in contact with the implant surface, and osseointegration is achieved. (C) Specimen of the 6-month complex, showing similar bone formation to the 3-month complex and clear increase in new bone formation at the implant interface.

At 2-month group of Implant/IP-CHA complex, newly formed bone mainly colonized the pores of the IP-CHA located in the vicinity of pre-existing cortical bone. In the central part of the IP-CHA, only in few pores, new bone tissue could be observed. At the interface between IP-CHA and implant no bone tissue, but only loose connective tissue was detected. In the coronal and middle portions of the specimens cortical bone were evident, while in the apical part marrow spaces. Newly formed bone could easily be distinguished by pre-existing bone as it appeared more intensely stained (Fig. 9A).

At 3-month group of Implant/IP-CHA complex, the pores of the IP-CHA located in the vicinity of pre-existing cortical bone and the ones in the central part of the biomaterial were filled by bone tissue. Bone could also be observed at the interface between the biomaterial and the implant mainly in the coronal and middle portion of specimens, while in the apical part very small and scarce trabeculae were present. The different intensities of the staining are less markedly distinguishable, owing to the process of maturation of the regenerated bone tissue (Fig. 9B).

At 6-month group of Implant/IP-CHA complex, the Implant/IP-CHA complex seemed to be integrated. Bone could be observed in the coronal, middle and apical portions of the specimens and especially in the coronal part many areas of contact between bone and implant surface were evident. Regenerated bone tissue could not be distinguished by the pre-existing one (Fig. 9C).

### Measurement of ISQ

**Figure 10:**
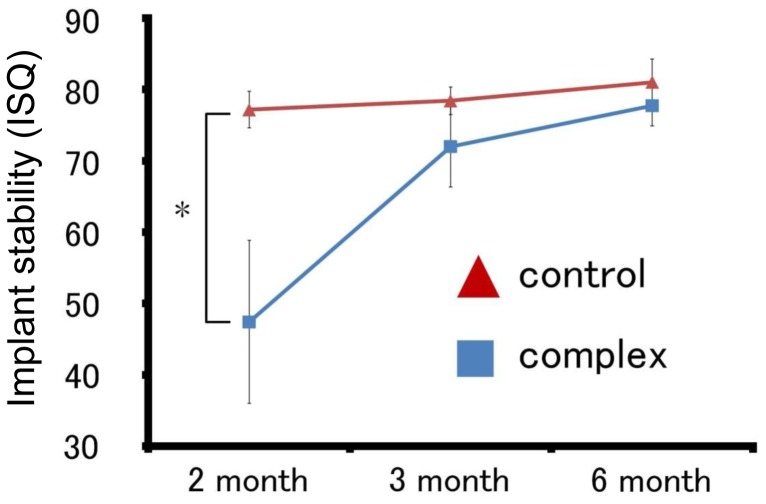
Mean ISQ of the Implant group and the Implant/IP-CHA complex groups. Asterisks indicate a significant difference with respect to the 2-month Implant as control group and the 2-month Implant/IP-CHA complex group (p<0.05).

Fig. 10 shows the ISQs of the Implant/IP-CHA complex and control implants, measured using an Ostell™ as the RFA. The ISQs of the Implant/IP-CHA complex was 47.4±11.4 at 2 months, 72.0±5.68 at 3 months, and 77.8±2.88 at 6 months. The ISQs of the control Implant groups were 77.2±2.57 at 2 months, 78.4±1.94 at 3 months, and 81.0±3.28 at 6 months. The differences decreased over time.

At 2 months, the ISQ of the Implant/IP-CHA complex was significantly lower than that of the control Implant. On the other hand, at 3 and 6 months, the Implant/IP-CHA complex ISQs were not significantly different from those of the control Implant group. The ISQs of Implant/IP-CHA complexes were significantly higher at 3 and 6 months than at 2 months (Table 2).

**Table 2:** Comparison of Mean ISQ for Implant/IP-CHA complex groups.

	Mean ISQ (SD)	Turkey test
**Complex: 2M**	47.4 (11.4)	
**Complex: 3M**	72.0 (5.68)	*P* = 0.0017*
**Complex: 6M**	77.8 (2.88)	*P* = 0.0064**

* between Complex: 2M and Complex: 3M.

** between Complex: 2M and Complex: 6M.

SD, standard deviation.

### Measurement of BIC

The BICs of the Implant/IP-CHA complexes were 2.18±3.77 at 2 months, 44.04±29.58 at 3 months, and 51.02±8.25 at 6 months. The BICs of Implant/IP-CHA complexes were significantly higher at 6 months than at 2 months (Table 3).

**Table 3:** Comparison of BIC for Implant/IP-CHA complex groups.

	BIC Mean (SD)	Turkey test
**Complex: 2M**	2.18 (3.77)	
**Complex: 3M**	44.04(29.58)	
**Complex: 6M**	51.02 (8.25)	*P* = 0.0356*

* between Complex: 2M and Complex: 6M.

SD, standard deviation.

## Discussion

In this study, the Implant/IP-CHA complex achieved both bone regeneration and implant stability.

The complex developed in this study could be built in two ways: incorporating the implant into the shaped IP-CHA, or burning apatite and implant simultaneously. The latter method, however, has some disadvantages. It is difficult to shape arbitrarily apatite, and titanium surface is formed oxide film because of high temperature burning. Therefore, it is considered that the former method is the most suitable to fabricate Implant/IP-CHA complex. The IP-CHA can be prefabricated into optimal forms and shapes using drilling, cutting or computer-aided design/manufacturing system [18]. Thus, IP-CHA can easily be formed into the desired shape using a cutting instrument. The shape of the screw-thread was evident in the SEM image of the formed IP-CHA (Fig. 4). Additionally, observation of the fabricated implant/IP-CHA complex showed that the area between the implant and the formed IP-CHA was mostly integrated (Fig. 5). Moreover, the distance between the threads of the implant used in this study was set at approximately 0.7 mm. At this size, it can be formed into an optimal shape by a screw instrument with same shape. These results indicate that the implant/IP-CHA complex may not only be applicable to this implant system but also to other implant systems used in the fields of dentistry or orthopedics. The requirements for a biomaterial used in bone regeneration are biocompatibility, good osteoconduction, mechanical strength, and availability of space for tissue and blood vessel regeneration. Moreover, the ability to use tissue engineering techniques, such as a creating scaffold for stem cells, or incorporating several growth factors is required. In this study, we used IP-CHA as a complex structure owing to its specific characteristics. Porous HA without interconnected pores has previously been used in situations where bone regeneration is needed [19], [20], [21]. While it possesses biocompatibility and promotes good bone formation, its osteoconduction is limited to its surface because of the lack of adequate connections between each pore [22]. It is widely accepted that pore sizes more than 10 µm in diameterare required to permit osteoconduction because the size of the nucleus in most mammalian cells is more than 10 µm [23]. In a clinical case, it was reported that bone ingrowth into porous HA without interconnected pores penetrated less than 300 µm from the HA surface [24]. Using such HA for an implant and HA complex would limit bone formation to the HA surface, and osseointegration would barely be achieved. In contrast, the IP-CHA has a systematic arrangement of uniform pores and almost all pores are onterconnected. The average diameter of the interconnection between pores is 40 µm, allowing efficient migration of bone-producing cells from pore-to-pore and colonization by blood vessels, which are essential for new bone formation.

In our previous study, we reported that IP-CHA was a potentially useful biomaterial for use as a scaffold; it has been shown to integrate bone marrow stromal cells and growth factors, and exhibited good osteoconduction in an animal model [13], [14], [15], [16]. With regard to histology, while bone formation clearly occurred at the IP-CHA implantation area in all Implant/IP-CHA complex groups, bone formation at the implant surface was only detected at 3 and 6 months. On the other hand, at 2 months, fibrous connective tissue or bone marrow was predominant at the implant surface. Considering the lengthy healing period, it may take more than 3 months to achieve osteoconduction throughout the implant surface. The IP-CHA of the complex has initial compressive mechanical strength equivalent to that of cancellous bone because it has a porosity rate of approximately 75%. While it is difficult to support an implant with this strength, mechanical strength is increased by the development of newly formed bone within the IP-CHA. In one study, the compressive strength of implanted IP-CHA was reported to increase steadily with bone ingrowth into the pores [6]. In this study, to evaluate osseointegration of Implant/IP-CHA, implant stability and bone implant contact ratio were measured. Measuring implant stability is considered to be an important method for evaluating the success of an implant [25]. The RFA technique has been shown to be an effective method of measuring implant stability [26], [27], [28]. This system measures ISQ using RFA, which measures emitting frequency via a vibration transducer attached to the abutment or fixture [29], [30].

The ISQ is given as a number from 1 to 100, where 1 is the lowest and 100 the highest degree of implant stability. ISQs of successfully stabilized implants are reported to range from 57 to 82 [27] and due to bone deflection, ISQ values as 90 or more are uncommon in clinical situation.

Additionally, histomorphological studies suggest that the bone-to-implant ratio and RFA values are related [31]. We measured the implant stability of all groups at 2, 3, and 6 months (Fig. 10). The ISQs of control Implant groups were found to be 70 or more, and significant differences were not observed in control implant groups. This is likely because the control Implants were placed in sufficient bone and initial fixation was obtained. The ISQ is known to increase in proportion to the stiffness of the bone-implant interface with that of the surrounding bone [32], [33]. Furthermore, no significant differences were observed between the ISQs of the control group and Implant/IP-CHA complex at 3 and 6 months. However, at 2 months, the ISQ of the Implant/IP-CHA complex was lower than that of the control Implant group or the 3- and 6-month complex groups. A significant correlation between the ISQ and bone cortical thickness was reported [34]. However, regarding ISQ associated with bone contact, there is a controversy. Abrahamsson et al reported there was no correlation of between BICs and ISQs [35], while, Blanco et al showed a there was positive correlation between the increase of ISQs and BICs [36].

In our result of BIC measurements of Implant/IP-CHA complex significant difference was observed between at 2 months and 6 months. This result seems to correlate between BICs and ISQs, however, the BIC at 3 month of Implant/IP-CHA complex was not significantly at 2month. It was due to that the BIC at 3 month had large standard deviation which suggested that bone contact formation was unstable.

On the basis of our histological evaluation, we consider that Implant/IP-CHA complex had stabilized and exhibited osteoconduction into the complex surrounding the implant at 6 months. A healing period of 2 months is not enough for osteoconduction to lead to adequate implant stability for the Implant/IP-CHA complex. The results indicated that implant stability was achieved using the Implant/IP-CHA after a 6-month healing period.

It is the authors’ intention to conduct further studies with experimental underloaded condition or mechanical properties to investigate detailed bone aspects in clinical model.

### Conclusions

Our results indicate that placed Implant/IP-CHA complex could achieve osteoconduction of newly formed bone and osseointegration at the implant interface. We concluded that the Implant/IP-CHA complex could be expected to achieve bone reconstruction and implant stability. The results of this study may also contribute to provision of increased knowledge of the development of more predictable bone graft materials.
